# Identifying and measuring land-use and proximity conflicts: methods and identification

**DOI:** 10.1186/2193-1801-3-85

**Published:** 2014-02-13

**Authors:** André Torre, Romain Melot, Habibullah Magsi, Luc Bossuet, Anne Cadoret, Armelle Caron, Ségolène Darly, Philippe Jeanneaux, Thierry Kirat, Haï Vu Pham, Orestes Kolokouris

**Affiliations:** UMR SAD-APT, INRA AgroParisTech, Paris Saclay University, Paris, France; Department of Economics, Sindh Agriculture University, Sindh, Pakistan; UMR Telemme, Aix-Marseille University, Aix-Marseille, France; UMR Metafort, AgroParisTech, Clermont Ferrand, France; University Paris VIII Vincennes Saint-Denis, Paris, France; CNRS, University Dauphine, IRISSO, Dauphine, France; UMR CESAER, INRA AgroSup, Dijon, France; Panteion University, Athens, Greece

**Keywords:** Conflicts, Methodology, Data base, Daily press, Litigation, Surveys

## Abstract

**Abstract:**

This text aims to present the methodology of study of land-use conflicts performed in recent years by a multidisciplinary team, and to reveal the methods of survey and data collection, as well as the structure of the resulting database. We first define the scope of our study by providing a definition of these conflicts, of their characteristics and motives, of the ways they manifest themselves and of the actors involved (I). We then present the methodology we have used to identify conflicts; it is based on a spatial analysis and the combined use of different data collection methods including surveys conducted by experts, analyses of the regional daily press and of data from the administrative litigation courts (II). Finally we present the resulting *Conflicts* © data base, with its tables and nomenclatures, in which the data collected in different fields are reconciled and analyzed (III), before providing a few examples of how this method can be used to analyze case studies in developed and developing countries (IV).

**Jel codes:**

D74; C83; K41

## Introduction

Though conflict analysis is inscribed in a long tradition of social sciences, at the first rank of which lies sociology (Lewin, 1948; Touraine, 1978; Stephenson, 1981; Simmel, 2008; Freund, 1983; Coser, 1982; Wieviorka, 2005), researchers and practioners have preferred to focus their attention on the questions of conflict resolution rather than on the analysis of conflicts and of their characteristics (Castro and Nielsen 2001; Jeong 1999; Fisher, 1997; Neslund, 1990; Owen et al., 2000), except in cases of armed conflict (Boulding, 1962; Diehl, 1991; Hensel, 2001; Starr, 2005). Yet, the growing concerns about the environment, the issues of sustainable development, urban sprawl processes and about questions about people's living environment have recently led to renewed interest for issues related to land use conflict, also called land use and neighbourhood conflicts or environmental conflicts (See, among many others: Humphreys, 2005; Deininger and Castagnini, 2006; Magsi and Torre 2014; Mann and Jeanneaux, 2009; Campbell, et al., 2000; Darly and Torre 2013a, b; Cadoret 2009; Melé et al. 2004; Dziedzicki 2001; Charlier 1999; Cadene, 1990).

Interest in these issues has grown in the fields of economics, geography, land planning as well as in sociology and social-psychology and has pointed to the necessity of analyzing conflicts, their occurrences, their impacts and main characteristics, more thoroughly. Such an approach obviously necessitates access to enough reliable data on conflicts per se, so as to be able to evaluate their number and volume, their role and impact, how they manifest themselves, what their causes or origins are and how they are solved.

Data about conflictuality is scarce and often incomplete for two main reasons. The first lies in the fact that little interest was shown in the subject until the years 2000. The second reason is related to the complexity of conflicts, which rules out the possibility of using only one representative variable. Indeed, land use conflicts find expression in various forms (tribunals, media coverage, violence…) which prevent one from making a simple representation and which explain why various disciplines are necessary to define them. A conflict that gives rise to analysis is a construct founded on information collected from different sources.

In light of this problem, a researcher wishing to study conflictuality must collect his/her own data (either they are focusing on developed or developing countries), and then analyze them (for more on the subject read Rucht and Neidhardt (1999) which describes the different stages that are necessary to this type of work). This is what we have done by developing a program of study on conflicts involving several teams of French researchers from various research institutes and universities. This program has successively focused on various issues pertaining of conflicts in natural, rural and peri-urban areas in developed and developing countries (France, Greece, Canada and Pakistan). Their contributions are based on analytical method adopted according to the nature and availability of data from different economies, and identification of land use conflicts, involvement of actors with different relations and network links.

This text aims to present the methodology of study of land-use conflicts performed in recent years by a multidisciplinary team, and to reveal the methods of survey and data collection, as well as the structure of the resulting database. The originality of this approach lies in its refusing to only use one particular source of information or one single innovative formula. The method we use to identify the conflicts, and which we present here, is complex and multi-dimensional. It involves a specific methodology that rests on the combination and triangulation of different sources and modes of data collection, and on precise protocols of data processing and of identification of conflict patterns, applied in each stage of the program. Following these protocols ensures that we achieve the most realistic representation possible of conflict within a given space or area. Our method borrows from other investigation procedures previously developed (See for example, Charlier (1999) based on press articles) and which we have transposed, improved and specified, but it also rests on innovative procedures (the use of litigation data and their treatment by means of software). It is based on social science analysis techniques (statistical inquiries, interviews, surveys, accounts, group follow ups…). It also consists in exploiting databases (such as the *Lamyline* database) or data provided by administrations (such as tribunals' judgments).

In some ways, our approach is not unlike that of other current projects of research on land use conflicts, in particular urban ones (see the research conducted in Canada (Trudelle, 2003; Joerin et al., 2005), in Netherlands (Leeuwen, 2010), or in Brazil (Observatorio Permanente dos Conflitos Urbanos na Cidade de Rio de Janeiro 2010). It is situated within a tradition that includes remarkable works conducted by teams of researchers located in various countries. Among these studies, three are, in our view, particularly significant, and have paved the way for our work by indicating the main difficulties involved in this type of research and by suggesting many solutions and possible paths for further research (Janelle, 1977; Ley and Mercer, 1980; Rucht and Neidhardt, 1999). Although we have not always drawn the same conclusions or adopted all the suggestions made by those authors, their works have been essential sources of inspiration. We owe a great deal to these studies in that they have helped us reflect on our multidimensional method of conflict analysis and avoid many pitfalls.

Two reasons explain why we have wished to present our method of analysis – which we have tested and improved over the last 7 years – to a wide audience of researchers and practitioners:

- We have wanted to show that it is possible to identify conflictual events and to deduce from them a general picture of conflictuality as well as a description of the characteristics of the conflicts that occur in a given area;
- We have also wanted to share our experience and encourage researchers to use our method, by responding to its associated criteria of intellectual property.

The structure of this article is as follows: we shall first present the scope of our investigations, placing particular emphasis on the questions of definition, origins and expression of conflicts. The second section of the paper is devoted to the presentation of our data collection method. We begin by describing the procedure of identification and diagnosis of the conflicts that occur in the area we have selected, and then discuss the identification method per se and more specifically its three foundation stones: the analysis of the daily regional press, of administrative litigations and the exploitation of surveys conducted by experts. In the third section we first present the *Conflict ©* database compiled from the information gathered. It addresses the questions of the construction and classification of the objects of conflicts, of the actors' profiles, of their utilization of land and of their arguments, successively. We end the article with a brief presentation of the results obtained from the exploitation of the database on conflictuality on selected case studies from developed to developing countries.

### Definition of the scope of investigations

We are interested in land use conflicts and conflicts over resources. In order to identify them, it is, first, necessary to propose an operational definition that will enable us to both recognize and capture conflictual elements and situations, and to classify them in such a way as to be able to trace the conflictual profiles of a given area.

Our research studies are conducted in rural and peri-urban territories. They pertain to conflicts and tensions related to public consumption commodities (air, landscape amenities and nature's functions), resources (water or energy), waste and pollution, as well as to the areas of location of individuals or activities and their neighboring areas.

We call conflict an opposition marked by an engagement or a commitment between two or several parties (the actors of the conflict), in relation to local material objects. These oppositions reveal local characteristics related to spatial dimensions (e.g. topologic dimensions, neighborhood and transport infrastructures) as well as to social and economic characteristics linked to the areas in which they arise. Land use conflicts are the results of the dissatisfaction of one part of the population with actions undertaken or planned by their neighbors, by private institutions or by the public authorities. They are the pointers of the innovations taking place in the different territories and of the resistance they generate, and also the ferment of new innovation phases. We do not consider it necessary to eliminate conflicts nor even to try and solve them at all costs, for they are the expression of the voiced opposition of parties that consider themselves injured. Conflictual events are phases of coordination between actors and a way of reintroducing new actors in the mechanisms of decision making and of creation of territorial development projects. Conflicts can often be considered as part of a trial and error method regarding public decision: if one decision is considered opposite to the needs and wills of local populations, it could lead to tension, and afterwards to conflicts.

### A local materiality

The conflicts we are interested in are distinguishable by their localized nature (i.e. territorial superposition of contradictory interests, rivalries between contiguous or neighboring areas), by the materiality of the objects that cause them or are concerned by them, as well as by the fact that they emerge in relation to differing land uses. Oppositions between individuals or groups pertain to concrete objects, to technical acts that are taking or will take place and imply concrete actions. These conflicts can have a strictly local component, or be related to questions that are more universal in scope. Whatever the initial situation, they can expand socially and spatially if they crystallize issues of a societal nature.

Land use conflicts have a territorial dimension. They rest on a physical basis: they take place between actors (sometimes, but not always neighbors) affected by a problem that has emerged and they develop around the use of localized support material or immaterial goods. They are inscribed in a geographic institutional framework, determined by both the actions of local and supra local authorities and by the rules they introduce. Indeed, *territoriality* and *unequal exposure* are central characteristics of land use conflicts. Environmental disputes are, first of all, *territorial* in nature to the extent that they involve the very unequal exposure of different territorial regions to environmental pollution, land speculation and development projects. Regions where the highest conflicts concentration is observed are also the regions where the densest concentration of risk-bearing facilities or building permits can be found.

Conflictual events are identifiable in relation to specific goods or pieces of land, i.e. the space within which the uses are in opposition. The cases studied in our research pertain to questions related to land, to territorial development as well as to water and its management, to the superposition of uses (production, tourism, leisure), to the development of economic industrial and port operation activities, to landscape and their evolution through urbanization and the creation of new equipment such as wind turbines, waste water treatment plants, waste management facilities, etc. More specifically, researches implemented in a country like France have been focused on conflicts dealing with the issue of urban sprawl and its consequences on speculation in farm land and forests (in particular risk of arson in peri-urban forests). In a Mediterranean country like Greece, forest fires can be mobilized as a strategy of land appropriation. Arsons can be used in speculative strategies to change land use destination, but can also indirectly provoke a local collective actions in favor of a stricter policy for the preservation of woodlands. Local inquiries paid also attention to the topic of farmland consumption and preservation of natural spaces.

The conflicts we have examined and their evolution pertain to the manifestations of oppositions to land modifying projects as well as to the effective emergence of constraints, pollutions and nuisances related to the changes that have occurred in the original space. Thus, the emergence of conflict is not necessarily related to the occurrence of a material event, but can also correspond to an expectation by certain categories of actors opposed to the project that that event will occur.

### The participants to conflicts: different actors and combination of actors

The individuals or organizations involved in land use conflicts can be divided into two large categories:

- Users of land and resources for productive purposes (whether or not they are the owners of the land in question and of their work tool): artisans and industrial entrepreneurs, farmers, herders and forest entrepreneurs, providers of recreational services involving the use of land;
- Users of land and resources for non-productive purposes (present on the land permanently - residents, hunters, sportspeople, hikers – or only present occasionally – tourists, secondary residents).

These categories of users find themselves involved in the tensions and conflicts, either individually or as parts of networks or groups of actors. Conflicts can involve users opposing one another (whether or not they pursue identical production goals), reveal oppositions between different categories of users. However, we consider that many actors can combine productive and non-productive functions, thus going beyond the simplistic dichotomy between the ones and the others. Indeed, they reveal contemporary social complexity and the various roles one person can play.

It is for this reason that we have chosen to base our work methodology on actors rather than on the uses they make of the areas considered. We also use the term "actor" so as to avoid referring to large categories of land users (residents, farmers, environmentalists, industries....) which are abstract and often account for only part of the reality and complexity of the actors themselves and of their relationships with others. Following the example of Janelle (1977) and of Ley and Mercer (1980) we also use the term "conflict participant" - which is the base economic and social unit of conflictuality - or conflict stakeholders.

### The motives of conflict

Conflicts arise from changes or projects of change, perceived by some actors as contrary to their interests or their wishes. The material expression of changes at the origin of conflicts pertains to several categories:

- Construction, deterioration or destruction of property, a landscape or infrastructure;
- Creation of a new production facility or expansion of an activity;
- Emission of external negative effects (diffusion pollution, odors, water drainage);
- Development of a property or piece of land;
- Access issues (restriction/exclusion, or opening/easement).

Nevertheless, the properties or facilities do not necessarily have to actually exist for a conflict to emerge. Conflicts can result from projects of construction, of implementation or extension of an activity, of development or of access modification, or from the emission of negative effects. In this case, conflicts are considered "anticipative" or "preventive". Indeed, disputes may arise between local residents or associations (acting individually or together), on the one hand, and the administration and applicants for permits to develop building projects or to operate activities, on the other. These disputes concern application for permits, above all, and come into play *a priori* (preventive conflicts), i.e., before the environmental risk-bearing activity or development project is actually launched. In this case, the dispute arises at the outset of the process. However, disputes may also arise when industrial operators or developers challenge administrative decisions or when local residents protest against the consequences of pollutions, nuisances and environmental impacts and the dispute occurs then *a posteriori* (curative conflicts).

### From tensions to conflicts

The distinction between tensions and conflicts is tricky to analyze. Indeed the emergence of a conflict follows an explicit engagement of the actors; an engagement which takes the form of acting out episode: threats, assault, actions at law, technical acts, and signs (forbidding access....). We shall call conflict any tension that turns into a declared confrontation, via the engagement of one or several parties.

*We then consider that tension between various parties designates an opposition without the engagement of the protagonists, whereas a conflict emerges with the engagement of one of the parties*. This engagement is defined by the implementation of a credible threat, which may take different forms:

- Legal actions
- Bringing the matter to the attention of the public authorities or of the civil service representatives
- Mediatisation (bringing the matter to the attention of the media, press, radio, television…)
- Assault or verbal confrontation
- Putting up signs (signs forbidding access, fences and gates…).

#### More or less apparent, individual or collective manifestations of conflictuality

Sporadic or recurrent, the tensions and conflicts related to the different land uses can manifest themselves in various ways:

- At inter-individual level: bad relations between neighbors, assault, recourse to third parties, retortion, reprisal;
- At a more general level: carried or handled by individuals (elected officials for example);
- Finally, at collective level, carried or handled by groups, particularly associations representing actors using land for non-productive purposes (these groups distinguish themselves from enterprises or large scale farmers or forest entrepreneurs in that they are characterized by a non-hierarchical internal organization and non-productive purposes), administrations, local or territorial authorities.

The strategies of the groups and individuals are closely related to the conflictual events. Highlighting them helps to explain the objectives and positioning of the actors at the onset of the conflict and in the ways to manage it. The way tension or a conflict is managed often depends on the goal pursued.

Tensions and conflicts have histories. Some conflicts pass away quickly, whereas others can last for long periods, with phases of more or less intense confrontation and stages of more or less latent antagonism. A phase of tension can last for a long time without turning into conflict, if the actors do not engage. During the period of tension preventive actions can be undertaken in order to prevent a conflict from happening. Nevertheless, it is important to note that it is perfectly possible for a conflict to arise without their having been prior tensions.

### Modes of prevention and management of conflicts

Although we make no hypothesis about the necessity to solve conflicts we regularly meet actors who seek to promote or implement modes of conflict resolution. The modes of conflict prevention and management can:

- consist of preventive actions aimed at promoting appeasement and at avoiding the occurrence of a conflict. These actions can be taken so as to promote inter-individual negotiation; they can consist in involving a third party representing land users, or in encouraging the actors to adopt a non-judicial route such as institutionalized mediation for example;
- merely consist of an arrangement between the actors involved;
- rest on a regulatory or legal techniques.

#### The solutions considered

Without going into an in depth description, we identify below the main types of solutions implemented:

- Technical acts;
- Compensations (financial, natural or technical);
- Land use planning;
- Eliminating the activity from the area in question or moving it somewhere else;
- Adjudication;
- Mediation by an insurance company.

### Methods of conflict identification

The identification and analysis of conflicts rest essentially, in our method, on following sources of data collection:

- The daily regional press (DRP);
- Surveys conducted by experts;
- Data from administrative litigation courts;
- Data through other sources are often added in studies on developing countries, where some of the previous relevant sources are missing;
- Internet (personal blogs and web pages)
- Geographic Information System (GIS).

Every source of data collection has its own limits. Only a small part of the conflicts (e.g. regarding the engagement paradigm previously identified) is treated by tribunals and a lot of opposition does not go on Courts. As far as the political traditions and the laws are different in each country, tribunals can play different and more or less important roles. The judgment can also be subjected to various interpretation or contestation, even regarding the administrative component. In the same spirit, there are severe limitations to the use of DRP. First, it is obvious that media behave differently with regards to the countries but also the regions or the areas within a given country. For example, they do not behave the same way in the north or the south part of France because of local traditions of public expression. It is also true that some media lie or forbid crucial events, or even at least euphemize about certain events, in order to hide a part of them or to reduce their importance. The same applies to experts opinions: they can lie, cheat, euphemize, or sincerely forget some crucial events. For all these reasons, we have chosen to base our identification and our analysis of conflicts on the triangulation of three main sources of data. Each one has its own limits, but altogether they provide a credible image of a conflict, at least regarding to the above definition.

A conflict is a social construction, which is identified by the observer and depends on the definition of conflict events and the types of observations. In our method, the identification of the conflict is based on a triangulation of the main sources of information (DRP, Surveys, litigation data or other data). It is by cross checking and comparing the various sources of information that an evaluation of the state of conflictuality in a given area is made. This job is performed by the experts of the team, on the basis of the main data. The status of “conflict” bears on at least two identifications in the main corpuses of data, for example in the daily press and regarding verbatim by local experts. The types of conflicts identified and the comparisons between different data bases aim at identifying several discrepancies between local situations; for example, DRP helps in revealing early oppositions to local projects whereas administrative litigations are related to well informed situations and revelation of infrastructure projects by mainly public authorities.

The identification of the conflicts, is however, preceded by the identification and diagnosis of the study area. Indeed, we focus on precisely delimited geographical areas and we collect and analyze the data pertaining to conflicts occurring within those zones. This enables us to describe with precision the conflicts and their evolution, and limits the number of possible occurrences.

### Identification and diagnosis of the study area

The area examined is always situated within an institutionally determined zone. Its geographical delimitation rests on that of the local public institutions, such as: communities of municipalities, conurbation comities, counties, regional nature parks, sub-regional constituencies.... The study area can comprise one or several of these administrative zones – several constituencies for instance. We shall only examine the events that occur within the chosen area. An exception is made for litigation studies. Because the amount of data on the rulings of secondary tribunals is usually too small and therefore not representative, we examine the district or region in which the study area is situated.

Once the study area is defined we perform an area diagnosis which must enable us to identify its main social and economic characteristics and its salient features and actors involved within it.

The basic diagnosis report, of approximately 10 pages in order to make comparisons, must comprise:

- A general presentation (location, the geographic morphology, history, social and economic dimension…);
- A presentation of the activities that depend on the resources available on the territory;
- The main territorial governance structures, particularly the institutions.

### The daily regional press (DRP)

DRP reaches in the hands of about 400 million readers around the world every day. It is the second most popular medium after electronic media, and therefore an interesting tool of observing voices of regional population. Furthermore, it has the twofold specificity of being the main medium of local news delivery and, for each daily regional paper, to often enjoy a quasi-monopoly in their circulation area. The DRP articles are an easily accessible source of information on pre, during, and post-conflicts, and complements efficiently the data found in other sources, through surveys in particular. The DRP provides relatively detailed information about local affairs, which is not the case of national newspapers (Rucht and Neidhardt, 1999; Mc-Carthy et al. 1996).

The work consists in gathering the information provided by a given daily newspaper, by examining all the issues published over a given period, of at least one year. The newspapers can be consulted online or in paper from, depending on availability. In the case of online consultation, the inventory can be conducted via the dissemination server providing access to the digitized editorial articles published by the main daily national and regional newspapers. The automatic keyword based search method is only used when information is sought about a particular topic, and not when a general inventory of all land use conflicts is needed. All articles are then displayed one by one, before being selected or not as part of our corpus. The articles quoted in the following sections were selected using criteria that have enabled us to differentiate the situations of tension from situations of conflicts (See Section Definition of the scope of investigations).

When the information contained in one article enables the researcher to identify the action, engagement and connection of an actor, or when the article provides complementary information about a previously described conflictual situation, it is indexed in the corpus using different variables:

- Its title
- Its date of publication
- The issue of the newspaper
- The section and page in which it appeared
- A very short summary of the facts described

Whenever possible a copy of the article is made (otherwise the summary replaces the copy). The list and the copies of the articles enable us, in laboratory, to group together the articles relating to the same conflict.

This process is not aimed at describing exhaustively all the conflictual situations. Rather, we deal here with a specific type of event, which is the one written about and reported to the public through the press. This source has important biases which rules out using it by itself. The press omits some events; it can have a tendency to euphemize, dissimulate, it can be partisan or controlled by certain interests. However, using the press has for several years been recognized as a means – in the perspective of an exhaustive quantitative analysis of conflict – of obtaining the "most complete account of events for the widest sample of geographical or temporal units", as Olzak (1992).

### Surveys conducted by experts

Interviews with experts provide information about the level of conflictuality. We aim to cross check this information with other sources, or to highlight conflictual events that might not have been brought to the attention of the courts or of the DRP. The interviews are aimed at identifying, in each zone, the dynamics of evolution of the rural and peri-urban areas concerned, at determining the types of conflicts and tensions relative to competing uses of rural spaces and at finding out the solutions implemented in terms of territorial governance.

We take great care of the fact that the experts interviewed have no private interest on land or other local resources involved in the conflicts, so that there are not personally affected by changes in land use for example. For the case studies quoted in next section the interviews were conducted with experts contacted beforehand by telephone, using a list of 40 to 50 people per study area. We choose to interview experts from various professional and associative fields so that a wide variety of opinions were represented. Each interview session (face to face) lasts between two and three hours. Each expert can be interviewed once, or several times when necessary (depending on the sufficiently required information). The respondents were interviewed with an open questionnaire so as to obtain as much information as possible, concerning conflicts and their evolution.

The questions may not directly be related to the conflicts. Indeed it has been observed that asking direct questions about conflicts generally leads the interviewees to refuse to answer. The interviewers present themselves as conducting surveys on local situations of governance, of actions and interactions between actors, sometimes with specializations depending on the interviews and the institutions they represent. The questions are always indirect: the interviewer must be trained to identify the conflictual elements of a situation. They must ensure that the questions in the grid presented below are all answered. The analysis of the conflicts is conducted later, in committee meetings.

This work enables us to gain a more thorough understanding of conflictual processes, to describe them and to identify their components:

- The materiality of the conflicts;
- The actors of the conflicts;
- The motives of the conflicts and how they emerge, which contribute to generating them;
- The manifestations of the conflicts, which imply various levels of symbolic or effective violence, the engagement of the actors ranging from petitioning to engaging in legal proceedings, or assault.

The 10-page report of the conflicts analyzed on the basis of the interviews with the experts lists the main conflicts that have occurred in the area considered, according to the interviews conducted. Quantification is impossible but the report provides insight into the perceptions of some of the key local actors on the conflictual process. A short one-page synthesis is provided with the report summarizing the main information obtained.

The interviews with experts cannot be our only source of information about conflicts for they present strong biases due to the method of analysis: the actors may have forgotten some elements; they may amplify or minimize, omit or lie about certain points. It is therefore absolutely necessary to complement them with other sources of data. Nevertheless, the interviews provide information that other sources cannot provide, and help us to gain insight into the dynamics of local alliances and oppositions, by enabling us to interact with the actors of the conflicts or with the observers of conflictual situations and of their development in the long term. We agree, here, with the conclusions of other studies about conflicts drawn on the basis of interviews with local actors, and more particularly with the conclusions of a study conducted for the World Bank by Deininger and Castagnini (2006) about the conflicts related to land property in Uganda.

Thus, we conduct semi-directive interviews.

#### Interview and interview analysis guide

The interview guide indicates the different items of information that must be collected in view of the analysis and exploitation. It does not provide with a clear description of the questions that must be asked during the interview. The interviewer must ensure that all items are discussed before ending the interview and must write a report. The questions to be asked and answered are the following; the order of the items is only indicative.

- Location (precise or extended)
- Spatial support of the conflict (punctiform or linear)
- Activities and their restrictions of use (productive, residential, recreational, "nature", exclusion, network infrastructures, public facilities)
- Number of actors (or groups) involved
- Actors (or groups) (by analyzing their degree of organization); distinguishing the actors who have an institutionally recognized mediation functional
- Origin/trigger of the conflict
- The causes mentioned
- Relation to time (duration, frequency)
- Relation to space (evolution of the space of concern during the conflict, by distinguishing if necessary, the places of litigation, the places where the conflicts take place and the places that are evoked by the actors)
- Forms of expression: (a) Verbal expression, individual expression (threatening mail, altercation, intentional damage of property, confiscation) collection expression (petitions, pamphlets, collective occupancy, marches, etc.). b) Departure (eviction, non-participation, passive refusal to obey a legal order or a summons, lasting refusal to participate. c) legal action (civil or administrative jurisdiction)
- Demands/claims declared or not by the protagonists (elimination or minimization of the nuisance – with or without a proposal of technical solution), demand for material or symbolic compensation, interruption of construction, etc.)
- Development (possible solution, the conflict continues, agreement, tribunals....)
- Public facilities possibly cited during the conflict (as a cause, as an element of context, as a solution)
- Types of arguments put forward:
  - effects on the personal living environment, versus activities, versus natural environment, collective, on the health of the individual or of the group, on the personal versus collective costs
  - on the principle: transgression of a locally accepted rule of a group, of an official law
  - Evaluation of the behavior of the actors who are statutorily assigned to intervene as mediator, as guarantors of the rules

We refer here to the list of local experts. They are obviously not all present on all the territories, but the goal is to provide information about each main category of the preceding Box and to achieve a balanced representation

#### The experts to be contacted on each site

*Local public institutions*

- Local elected officials: mayors of municipalities and other elected representatives (General councilors…)
- Directors/managers of inter-municipal services/facilities
- Economic director of inter-municipal planning
- Chairman of an inter-municipal body or of specialized committees (environment, tourism, agriculture, commerce…)

*Institutions for the protection of the environment or organizations of nature users*

- Regional Directorate for the Environment (environmental protection agency)
- Local associations for the protection of nature or for the environment (water, hikers or other outdoor sportspeople)
- Federations of hunters and fishermen and their local associations
- Environment and Energy Management Agencies

*Forestry and agriculture related organizations* (Specific attention is paid to this area of activities, and particularly on agriculture)

- National/regional forests offices
- Federation of Forestry Owners
- Local Land development and management bodies
- Chambers of agriculture
- Planning departments and agricultural economics services
- Federation of Farmers Unions
- Regional directorates (planning, economic services, agriculture and forestry)
- Farmers (in person)…
- *Socio-professional representation*
- Chamber of trade
- Chamber of commerce and industry
- Entrepreneurs clubs
- *Infrastructure planners*
- Water agency
- Agency for electrification and water management
- Agency for highway/motorway construction
- Land Development and Rural Settlement Associations
- Land acquisition and relocation committees
- Department of railways
- Local construction agencies
- *Other State services*
- Sub-prefectures (general secretaries or attached functionaries)
- Economic services
- District Court clerk
- *Other sources of information*
- Local press journalists
- Notaries
- Judges…
- Police department

### Analyses of litigation rulings

Administrative litigations should be collected from respective courts for statistical analyses. Therefore, statistical analysis of judiciary sources aims at examining the way in which legal rules are mobilized in land use conflicts, using a study of litigations between the parties involved in conflicts. This analysis targets a specific category of conflicts: those that have taken a specific path and have led to legal proceedings.

The inquiry on conflicts, through the quantitative study of litigation, is an empirical methodology that has its own limits and opportunities. As for the limits, the research in legal sociology usually concludes, regarding empirical data, that the resort to courts is always intrinsically marginal, may the phenomenon be observed in developed or developing countries. The successive social acts that consist to get informed on its rights, to choose a legal advice to solve the dispute and to go to the courts constitute different stages that select the people involved in conflicts. But at the same time, even though the actors reluctant to settlement and going to courts are a small part of the whole set, this tiny fringe is likely to be a mass phenomenon concerning a huge quantity of cases that can be studied statistically. Furthermore, the study of this small fringe of litigants can inform us about non judicial settlements: for example when cases are filed but dropped before they are adjudicated, it may be a sign that the resort to courts was just a mean to open a negotiation.

In some developing countries, this selective dimension of litigation is surely more acute. Cultural and financial barriers for the access to courts, the likelihood of corruption among the judges, the weak probability of implementation of court decisions, given the failure of public administration, are disincentives for a judicial strategy. Nevertheless, if the social phenomenon of litigation is quantitatively less important in those countries, our researches (conflict over water dam in Pakistan) and other empirical inquiries (for example, the world survey of Antonio Azuela for the Lincoln Institute about the conflicts over land evictions) (Azuela and Herrera-Martin, 2010) show that the dynamic of the process can be favorable to a development of judicial strategies. In that case, the research will focus more on evolutions that indicate an increase of legal consciousness than on the intrinsically low level of judicial disputes on the short term.

In France, disputes concerning activities which bear environmental risk or are likely to impact farmland or preserved natural spaces are adjudicated by Administrative Tribunals. These frequently involve claims initiated by associations and local residents against projects which have been granted permits by administrative authorities but whose impact is alleged to be harmful to the health and well-being of the local environment and community.

The various forms of actions at law have also been the objects of fruitful investigations in matters of infrastructure development or urban planning. The study of the practices in penal law in this field reveals the maneuvers possibilities that exist at all stages of the treatment of the offence, and the possible solutions: by the administrative authorities that detects the offence, by the prosecutor who decide to not prosecute, or by the judge who decides to pass an alternative sentence. They also highlight the diversity of the origins of the offence reports submitted to the legal system, depending on the priorities determined locally by the administrations concerned – targeted prosecution campaigns over a given period so as to solve a local problem – in a field where offences are much more often reported by booking officers than by the victims of the offences (Struillou, 2004).

With the exception of rare studies targeting specific associative actors, (Leost, 1998), the statistical studies undertaken on the basis of data from administrative jurisdictions are almost non-existent, in the field of land-use litigation. These sources are only used in the framework of general statistical studies on the activities of administrative tribunals, which, however, provide a useful basis for conducting more targeted surveys (Barré et al. 2006).

Therefore, the analyses of conflicts based on the observation of legal and administrative litigations are conducted at small administrative unit (*department, district, division etc.*) level. This choice is justified by two arguments: on the one hand, that particular administrative unit is the territorial unit of reference for many actors, whether they be public actors in charge of land use regulation (prefect, non-central government services), or para-governmental and private actors: Associations for the protection of the environment generally act at these administrative levels; the same applies to chambers of agriculture or the associations of hunters and fishermen; on the other hand, court rulings generally state precisely where the problem is situated at municipal level (private litigations, applications for annulment of municipal decrees or deliberations) or at departmental level (applications for the annulment of prefectoral decrees).

Land use and environmental conflicts consequently reflect the way this administrative activity operates. Knowledge of the inner workings of this system on a normative and empirical level is imperative in order to understand the logic behind the litigation. In the case of administrative justice, this prerequisite seems easier to meet than in other fields, like criminal sociology, or the sociology of civil disputes, to the extent that the dispute can be traced to administrative decisions and activity for which there are more likely to be written entries. This may not be the case for private law documents or contracts, so it is easier to obtain data on the frequency of claims in the case of administrative justice. It is therefore with regard to the intensity of conflictual activity must be assessed.

In developed countries, especially in France that the corpus of court rulings is built using the *Lamyline* textual legal database which comprises the complete texts of the rulings made by the Court of appeals and Supreme Courts. More precisely, this database comprises all the rulings made by:

- The State Council (highest administrative jurisdiction in France) since October 1st 1964,
- The Administrative Appeal courts since January 1st 1989,
- The Court of cassation (highest civil jurisdiction in France) since October 1st 1959 (excluding the rulings by the Criminal Chamber which are included from January 1st 1970 onwards),
- The Court of Appeal since January 1st 1982.

The software has a search engine that enables us to use the Boolean operators and those of several French case law data bases. The search for rulings by the four levels of jurisdiction defined above is conducted from the following case law data bases:

- State Council
- Administrative Appeal Courts
- the Court of cassation
- Appeal Court

Contrary, in the developing countries it is often difficult to collect the statistics from administrative courts, because of the absence of case law databases. Thus cases of the courts can be collected by searching manually from the yearbook records in their libraries, which is of course a time taking process. While in some developing countries uncooperative behavior of the administrative courts is discouraging the researchers^a^. Although the case law hierarchy is not the same in the entire developing world, it follows more or less the following structure:

- Supreme court
- High court
- Session court

Obviously, only part of the land use conflicts has been analyzed: those that have been dealt with by Courts of law, which limits the number of conflicts selected and examined. Indeed, the "passage through court" is the result of a selection using various filters: an individual's refusal to negotiate, an administrative authority's refuse to regularize… or on the contrary the intention to use the tribunal as a lever for starting stalled negotiations or for conducting them with a more favorable balance of power. These elements contribute to giving the conflicts that have been dealt with in court a profile whose specificity must be discussed in the interpretation of the surveys' results, without however considering that they represent all the conflicts that occur in the various territories.

### Data from other sources

The identification and listing of the conflicts are performed on the basis of the data from the three sources described above and on their comparison.

Nevertheless, other secondary methods of analysis can be added to this initial list, especially in developing countries or less developed countries, where some of the previous sources of data are missing. This can be particularly true for litigation data. These additional methods are aimed at conducting a more precise analysis or at specifying such or such dimensions of conflicts.

We shall, here, content ourselves with mentioning them. They are respectively:

- Sectoral analyses: for example, analyses targeting the farming sector or land property issues;
- In depth analyses carried out by researchers/experts in specific disciplines: for example, interviews of actors by sociologists, or monitoring of meetings or postal questionnaires administered in the context of research in social psychology;
- Study of particular situations: for example, study concerning a river, or farming facilities or land takes for road and water reservoir construction.
- In depth analyses of digital aerial photographs and remote sensing images through Geographic Informative System (GIS) of the territory; for example if the changes over a piece of land are being monitored in case of superposition of uses and its impacts;
- Analyzing personal blogs and web pages through internet, which is growing source of information on opinions and expertises in the developing countries.

Inquiries implemented in Greece combined different sources, from local newspapers to online websites supported by environmentalists associations (“observatory of natural spaces”). The latest source has the advantage to cover a large period (2001–2011), but is characterized by a selective approach, since data are furnished by local activists. Other sources complete the research methodology as follows: interviews with local experts (journalists, lawyers…) and key stakeholders like planning agencies, political leaders and elected officials, additional events collected through other web platforms (blog sites of environmental activists). An important source of documentations was also constituted by political materials like programs of local parties for municipal elections.

For analytical methods of the case study from Pakistan (presented in next section), data have been collected from various secondary sources to crosscheck the results. For example, the information has been collected from literature published by public and private organizations, on the case study perspective. The data have also been collected through aerial photographs and remote sending images, through satellites which were treated under GIS in order to examine land cover changes and impacts of the superposition of land uses due to decision for infrastructural projects by the public authorities over existed economic activities in the region. This technique of research became reliable after development in technical software. Moreover, data also collected after in-depth analyses from personal blogs and different web pages, these sources of information are very promising for future researchers of land use conflicts due to huge development in internet in the developing countries.

### The Conflict © database

The scope of our method is rather large, and we intend to provide it as an opportunity to studying land and resources conflicts in developed as well as developing countries. However, we have built a data base related to our own investigations, especially in France (13 case studies in different areas) and Pakistan. This data base can be expanded or reproduced for other types of investigations, in other countries or regional areas, given its wide scope and the general character of the contained items. We will present it in in broader terms in the following paragraphs.

In the context of the research conducted on conflict, using the relational database responds to a twofold analytical need. The aim, first of all, is to create data that can be exploited quantitatively, using the documents and survey results: the coding operations performed on the basis of the source documents are aimed at quantifying the phenomena of conflictuality, which will be analyzed and possibly examined from the perspective of the profiles of the areas concerned. But this database must also enable us to conduct a comparative evaluation: comparison between the sources used (court cases, newspaper articles, questionnaires), and comparison between the different areas surveyed.

The comparison between the different sources rests on the database' structure, which is designed in such a way as to be able to compare them. Besides the variables relative to specific observation contexts (type of rules used in the cases, number of press articles discussing a particular subject) some common variables have also been defined. The researchers conducting the coding operations must translate the specificity of their material in these cross sectional categories – described in detail below – relative to the types of conflicts observed, to the actors involved, or to the uses and arguments discussed.

### Database structure and categories of conflicts

**Figure 1:**
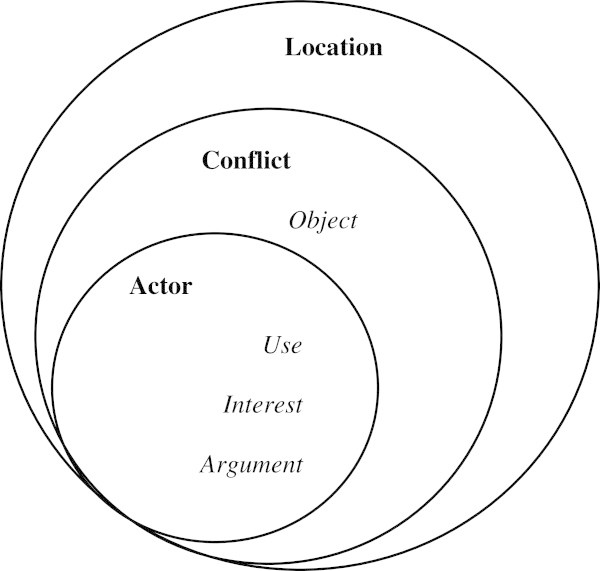
**Simplified graph of the database' structure.** The circles represent the relationships of inclusion between the tables. We have noted in italics the tables that are dependent on the "conflict" and "actor" tables.

The overall structure of the database can be schematized in the Figure 1 below. The data base incorporates three main data tables, that is, in order of inclusion:

- A table containing the variables relative to the geographic locations of the conflicts (in relation to a municipality, a community of municipalities, a *département* or a district);
- A table indicating the variables describing the conflicts per se, that is, the cross sectional categories – which are identical whatever the source of the data, and the categories relative to a context of observation (The legal categories defining, for example, the nature of a request made to a jurisdiction);
- And finally a table providing information about the profile of the actors involved.

Four other dependent tables must be mentioned: the description of the conflicts' "object", as well as information about the actors concerned: what the actor involved in the conflict uses the land for, the arguments the actor uses, and the interests that motivate the latter's engagement in the conflict.

The difficulty of studying conflict in one territory using different types of sources has to do with the identification of comparable analytical categories, whatever the observation point selected. This difficulty is crucial in the analysis of the requests sent to tribunals, in so far as those requests are expressed in a constrained language, that of the legal categories. Moreover, the enumeration of the criteria defining the contours of a conflict as a homogenous entity is a methodological demand which becomes particularly acute when exploiting press articles. Thus, while "formatting" the actions at law as clearly identifiable cases naturally leads us to choose "case" as the unit of measurement of the "conflictual" event, it is the "article" that is used as the measurement unit for the analysis of the press, when reconstituting the event after it has occurred.

Furthermore, a common definition of categories that cross the analysis of actions at law and that of the press, has to be found in order to enable the researchers to use a common language of description of conflicts. This does not exclude the necessity to define, for each type of observation material, specific variables relative to the source used. Thus, the nomenclature of requests, used by administrative court clerks for example, has been used for internally describing the different types of actions at law. Similarly, some variables are only meant to be used in the framework of an analysis of the press (number of articles devoted to a particular conflict). We present here the main components of the database, which is described comprehensively in several internal documents (for example, Galman and the participants of Conflict Program 2007).

The trickiest variables to define are, naturally, those that pertain to the different objects of conflict (see below). The detailed examination of the categories defined reveals the combination of several modalities of definition; complexity inherent to the expression of the forms of conflictuality. Indeed, the "objects of conflict" refer, depending to the cases, to:

- more or less clearly territorialized economic activities, whether they specifically involve using natural resources (agriculture, extraction of underground resources), location of production activities (industrial production, production of energy, waste treatment), or activities related to the presence of amenities (tourism);
- types of legal authorizations granted by administrative authorities, in as much as they correspond to land uses as defined by law;
- forms of social relationships marked by geographical specificities: Neighborhood relationships for instance.

#### The objects of conflicts: the categories used

- *Accessibility and easement*
- Right of access and passage
- Occupancy/parking
- *Facilities classified for the protection of the environment*
- Quarries, borrow pits
- Salvage/recycling, storing, waste treatment
- Salvage/recycling storage of materials (cars, tires…)
- Production, storage of chemical substances
- Other regulated industries
- *Site rehabilitation*
- Mining site
- Industrial production site
- Storage site
- *Service activities*
- Tourism, leisure
- Transport, Fuel distribution
- Trade, retail, advertising
- Road, rail, sea, air transport
- *Agriculture, forestry, fishing*
- Farming
- Forestry
- Fishing
- *Public utility infrastructure*
- Airport, railway, road, harbor infrastructure
- Construction of water reservoir
- Production – energy transport
- Telecommunication infrastructure
- Public facility
- *Management and Conservation of Natural Areas*
- Hunting/fishing
- Water quality management
- Soil quality management
- Air quality management
- Landscape, wetlands
- Conservation/ management of the Fauna/flora/biodiversity
- *Urban planning/development operation or document*
- Camping sites
- Urban development document
- Right of pre-emption
- Public domain occupancy
- Land restructuring
- Risk management areas
- Natural areas
- Management of natural areas
- Construction/extension of agricultural buildings
- Construction/extension of residential building
- Construction/extension of commercial building
- Construction/extension of thoroughfares
- *Neighborhood*
- Neighborhood nuisances
- Declaration of co-ownership
- Theft, damage, attack

### Profiling the actors engaged in conflicts: categories of actors, land uses, arguments

The analysis of the actors engaged in conflicts is summarized with great care in the grid of the variables used. Once the credible engagement of an actor has been identified, one can, indeed, distinguish the actors that are at the origin of this action (the protesting actors) from those which this action targets (the disputed actors).

As stated in point 1.2., the typology of the groups of actors that we have used for the database is founded on a distinction between users of land and resources for production purposes, whether or not they are the owners of the land (farmers, forest entrepreneurs, artisans, industrial entrepreneurs, providers of recreational services…) and users of land and resources for non-productive purposes, whether they use the land in question occasionally or continuously (residents, hunters, fishermen, sportspeople, hikers, tourists, secondary residents…) (See below). If a local authority body, or a State department is, for example, user of resources (in the case of well boring for water for municipal consumption, or of public road maintenance by the public services), we shall consider the actor concerned as a "representative", considering that during a conflictual confrontation, s/he will refer to the authority which his role as representative confers upon him/her, and as defender of collective interests.

#### The categories of actors involved in the conflicts studied

- *Farming or equivalent occupation*
- Farmers
- Irrigation operators
- Forest entrepreneurs
- Fish farmers
- Herders
- *Industrial actors*
- Artisans
- Industrial entrepreneur (mining activities)
- Construction and civil engineering firms
- Industrial entrepreneurs in the manufacturing industry
- *Actors of the market service sector*
- Transport enterprises
- Energy suppliers
- Providers of services related to waste management
- Providers of services related to water management
- Real estate developers, planners
- Actors of the hotel industry
- Other market services
- *Associations*
- Ass. For the protection of the environment (national or worldwide)
- Ass for the protection of the local environment
- Hunters and Fishermen's Association
- Political party
- Other associations
- *Local public authorities*
- Region
- Department
- Municipality
- Public Intermunicipal Cooperation Institute
- Regional Natural Parks
- Local Administrative body (water agency, Committee for the protection of natural sites and monuments)
- *National Public authority*
- Minister
- Prefect
- De-concentrated State services
- Judicial authority
- *Elected representatives*
- Municipal, departmental, regional representatives
- *Professional organization*
- Trade unions
- Consular chambers
- *Individuals*
- Permanent residents
- Temporary residents
- Owners syndicates
- Tourists, non-sedentary population

Once the actors of the conflicts are identified, we describe the controversial or conflict generating land uses that is the land uses the spatial consequences of which the objecting parties consider as sources of constraints. The distinction between actors and uses requires the taking into account, through standard observation, of the fact that one user can use land for several purposes: A farmer can also be a herder, a hunter, or a defender of nature; an industrial entrepreneur may also be a hiker; a resident may in the framework of his/her work conduct polluting activities…

Different configurations of opposition emerge, including between actors making the same use of land. Particular attention is placed on the local public mechanisms that are liable to exacerbate or crystallize certain tensions into conflicts.

#### The categories of land uses identified for the analysis of conflicts

- *Creation of infrastructure*
- Production and transport of energy
- Road, railway, airport infrastructure
- Waterway transport infrastructure
- Waste management
- Water supply
- Public buildings
- Telecommunication
- Advertising infrastructures
- Recreational/tourism infrastructures
- *Production of services and exploitation of infrastructures*
- Tourism, hotel and catering industry
- Telecommunication
- Air, road, railway, river, sea transport
- Production and transport of energy
- Trade, advertising
- Sanitation
- Domestic waste transportation and management
- Water catchment, treatment, storage and supply
- Management of hazardous waste and materials
- Utilization of buildings housing public services (functioning)
- Exploitation of recreational or tourism infrastructure
- *Agricultural, fishing and forest production*
- Agriculture
- Aquaculture
- Breeding
- Professional fishing
- Forestry
- *Industrial production*
- Mining
- Manufacturing
- Site rehabilitation
- *Recreational and touristic use*
- Hunting, fishing
- Hiking, motor sports, non motor sports
- Tourism’s events (cultural, musical, etc.)
- *Residential use*
- Construction/extension of social housing
- Development of poor housing
- Residential use
- *Conservation and management of resources*
- Fauna, flora, biodiversity
- Surface and underground water
- Site, landscape
- Ground
- Heritage
- Risks

#### Absence of a characterized land use

Just like the distinction between actors and users, the identification of specific registers of argumentation is based on the hypothesis that some categories of actors can, depending on the conflictual situation, use specific arguments. The capacity for technical and legal argumentation can for example depend on the degree of collective mobilization which a given conflict gives rise to.

An important cornerstone for the analysis of conflicts consists, indeed, in highlighting the registers of argumentation used by the actors. Argumentative openness implies that the (individual or institutional) actors diversity their registers both from the point of view of values and that of rules, so as to lend as much efficiency as possible to their protest movement. It can perfectly well result in contradictions when the same actor puts forward both the individual's interest and the general interest. But beyond this classic opposition, these argumentative strategies appear to be more complex when using the "general interest" argument requires the mobilization of distinct norms, which are often revealing of contradictions in the law itself (Lascoumes, 1995): thus, in the context of a protest against an urbanization project, the principle of controlled development of land and the argument in favor of housing development are used in turns; both categories of general interest are recognized by legislation.

#### Registers of argumentation: categories of analysis

- *Scientific and technical argumentation*
- Protection of ecosystems
- Infrastructures and equipment
- Risk evaluation and management
- Socio-economic argumentation
- Economic interest
- Sustainable development
- General local, regional or national interest
- *Reference to private land rights*
- Access
- Ownership
- *Responsibility*
- Private responsibility
- Public responsibility
- *Quality of life*
- Perception of risks
- Living environment
- Insecurity of individuals and property
- *Values*
- Modernity
- Tradition
- *Respect of the law and regulations*
- Risk prevention
- Other

### Constitution and exploitation of the data originating from the daily regional press

After collecting the data from the DRP, the inscription is made in the data base using the following schemes. For each conflict identified, we include in the database a series of descriptive variables that groups together the following elements:

- A brief summary of how the conflict developed, as described in the relevant article or series of articles,
- The spatial environment of conflictual events, the facilities - being used or subject to usage restrictions – that are blamed as sources of constraints,
- The resources that have been modified, and the usage or activities affected by these modifications (reconstituted using the provided arguments),
- The opposing actors and their modes of engagement in the conflict,
- The geographic location of the goods/resources affected by the controversial facilities^b^.

After analysis, we compile a 10-page long data sheet about the conflicts reported in the DRP. It lists the main conflicts that have occurred in the zone considered and classifies them according to the number of times they have been mentioned in the press. This helps us to obtain a picture of conflict, and of the media impact of the different causes of conflicts. This datasheet is accompanied by a one page synthesis summarizing the main information obtained.

### Constitution and exploitation of the data originating from litigation rulings

Once the database originating from litigation court ruling is compiled, it is analyzed statistically and lexically. The decisions are coded in such a way as to constitute a database integrated, first, into an Excel spreadsheet and then incorporated into a data processing software system (4D). The variables and modalities are defined using the above mentioned grid of analysis of conflicts also used for the interviews with experts and the DRP.

That corpus is exploited through the sequential and cross comparison of the significant variables, and through the analysis of the frequency of the legal references made. This analysis is completed by the textual statistical analyses performed with the software ALCESTE, so as to identify, through the language used in courts, the local specificities of the conflicts. These lexico-metric tools have only been used in the framework of the analysis of legal documents, which are well suited to this type of analysis due to the highly formalized structure of the argumentation. Without describing in detail the modalities implemented and the results obtained with this tool (Kirat and Torre 2004), it is useful to specify its scope as a component of a wider methodological approach to conflict analysis. The analysis of lexical statistics is justified by the fact that the language used in the court rulings is the product of the legislative or regulation texts used by the parties or the judge; this terminology is used to describe objects of protest or demand but also to express logics of action and goals (obtain the re-establishment of a right-of-way easement, the cancellation of a building permit in a zone of ecological interest, or the cancellation of a public inquiry conducted in the framework of a project of creation of a Classified facility for the protection of the environment). The lexical analysis is one of the elements of the study of the arguments mobilized by the actors. It enables us to show that one generic type of conflictual factors does not give rise to one unique and universal model of action in administrative courts. In other words, the actors seeking justice do not behave in a homogenous manner in all the *cases* studied.

The use of ALCESTE is not a technique for a-priori hypothesis testing, but for exploration and description. The program generates an empirically based classification of text units according to the pattern of co-occurrences of word tokens within these units. The lexical analysis software operates by dividing the corpus in elementary context, that is to say, a group of words identified by their length and punctuation. The similarity of these sentences is determined by reference to the similarity of the words used. A descending hierarchical classification distinguishes several classes of context units, and measures the closeness or distance between them. The classes identified constitute a basis for a principal component analysis (PCA), which is then used to realize graphical projection structured by the two most significant vectors. The researcher then begins to make interpretations based on the output from the program. It facilitates the discovering of the salient meaning structures and implements mechanisms for an independent analysis of the meaning of words, in order to get a statistical ranking of lexical statements in a given corpus.

The search for court rulings is performed by crossing the name of the departments selected with several keywords, defined in such a way as to cover as comprehensively as possible the scope of legal questions in which the land use conflicts can be formulated.

#### The search keywords used for the compilation of a sample of cases

- Easement
- Hunting or hunting right
- Building law
- Farm laws and hunting
- Farm laws and protection of nature
- Co-ownership or nuisance
- Directive 92/43/EEC
- Land application or breading
- Fauna and flora
- Classified facility
- Disturbance
- Nuisance and olfactory
- Nuisance and noise/sound
- National Natural Park or Regional Natural Park
- Passage and (hiking or Moto or quad)
- Rural land reparcelling
- Normal nuisance or neighbourhood nuisance
- Wetlands or swamp or bog

The judgments made in cases that occur locally at departmental level can be identified thanks to the effect of standardization of court decisions: the latter must include the address of the parties involved in the trial. Moreover, most decisions include the name of the study zone.

For example, the text of a judgment rendered by the French administrative litigation court is systematically made of four sections. The first states the identity of the petitioners, the nature and date of issue of the contested administrative act, and the administration that has issued the act, which is therefore sued in the litigation. The following section describes the judge's answer as to the means of form used by the different parties, that is to say the arguments relative to elements of the procedure (receivability of the request, the petitioners' right to act, etc.). The third section contains the judge's answer concerning the substance, that is to say the arguments used to oppose or to defend the administrative act itself. The fourth and final section evaluates the sanctions and compensations to impose depending on the ruling given.

At the end of this search, the corpus is reviewed so as to eliminate duplicates; indeed strong redundancy between the decisions found using different keywords can occur. The non-relevant decisions are subsequently eliminated, such as for example, the judgments concerning hunting accidents, or those relative to cases occurring in departments other than that where the parties reside…

Once the data is analyzed a 10-page datasheet on the conflicts identified through the exploitation of the litigation courts' data is compiled. It describes in detail, for each type of jurisdiction concerned (administrative and civil), the categories of requests that are the most frequently submitted to tribunals, and also indicates the main argumentation strategies observed according to the categories of actors (what corpus of rules is mobilized by what type of actor?). The question of the "outcome" of the conflicts, sometimes difficult to evaluate when analyzing the press, is here systematically interpreted, in so far as with the exception of abandonment or discontinuance of a procedure, a case judged on the basis of its substance gives rise to a decision that will oppose a "winning" party to a "losing" party. Thus analysis of the "rate of success" per category of conflict and per type of actors is therefore an important component of this synthesis. As a matter of fact, as in the case of the press and the interviews with experts, a one-page synthesis summarizes the main information obtained.

### Added local socio-economic data

In addition to these elements we include in the database information relative to the area and municipality concerned and useful for understanding the local context in which conflicts emerge. These data are of two types:

- First of all, they are socio-economic variables describing the profile of a territory from the point of view of social dimensions (tax related data, proportion of social housing…), of environmental issues (percentage of farm and natural lands, of areas protected for their heritage value), of the demographic dynamics (migration, population pyramid), etc.
- A second group of data provides information about the different administrative decisions that are liable to give rise to opposition: Building permits granted by mayors (data on the authorizations granted by the Regional Directorates of Infrastructure), or permits issued by the prefectures in accordance with regulations *concerning classified facilities.*

Both types of data refer to two levels of explanation of conflictuality: The first, immediate, level of explanation helps us to evaluate a rate of conflictuality in relation to an activity of reference that generates controversy. Thus, intense conflictuality concerning urban development generally reflects a highly dynamic construction market. The succession of protests related to pollution issues is often the consequence of an area being highly exposed to nuisances because of the important number of classified facilities. However, the intensity of this conflictuality can be, in relative values, higher or inferior to that of the activity of reference. Using data on local social and economic background enables us to test hypotheses on the "long term" relationship between the dynamics of a territory and the modes of conflictuality, and to incorporate social and human dimensions. Thus, the level of conflictuality often proves higher in areas where the levels of income and education are high, which implies that the population mobilized is well informed and educated.

### Illustration of data crossing using the conflict method. Examples from developed and developing countries

The use of our methodology of analysis, and of the data contained in the *Conflict* © database led to several works^c^ (some of them, marked by asterisk * are presented in the bibliography), performed on a few case studies, and to an increasing number of publications, quoted above. Our aim, here, is not to enter into all the details of these studies, but to provide examples of studies based on different areas, in quasi opposite situations. Thus, we have chosen two examples, based on two case studies that have been studied extensively by our teams, in order to provide information on the way the method and the data base have been used but also about the options provided by them in front of different situations. The two case studies (the Greater Paris region and the Chotiari reservoir case in Pakistan) are rather different in many ways:

- The Greater Paris region case study is located in a developed country, in a very densely populated area, in a peri-urban and very rich region, involving rural and urban inhabitants, conflicting for scarce resources;
- The Chotiari reservoir case study is located in developing country, in a sparse area, in a rural and very poor region, involving local users of land in a conflict against the loss of their land and natural resources.

Thus following subsections are devoted to the presentation of the dynamics of land use conflicts in these two areas, involving reflections on the use of our method in developed and developing countries, and about the possible adaptations to changing situations. We have also highlighted the different results provided by the use of various data, and their comparative utility.

### Conflicts in developed countries. An example of analysis of conflictuality in the greater Paris region

The research conducted using our methodology in developed countries has led to studies of several sites located in rural and peri-urban areas in France^d^. They have resulted in several publications that jointly or alternatively use one or several of the aforementioned sources. They are of great importance with regards to the French case, because there is an ongoing debate on the role of land-use and proximity conflicts in rural and peri-urban areas. Namely: which types of conflicts occur in these zones? Are they related to public or private actors? Are they legitimate or are they part of the Nimby phenomenon? Can they be considered as signals of public decision failure? Do they prevent local development?

An illustration of how we have used the data collected using our method, performed by using the *Conflict* © database, is provided through the analysis of the levels of conflictuality in the Greater Paris Region, one of the areas surveyed (Darly 2009; Pham and Kirat, 2008; Pham et al. 2012), and, more particularly, through a comparison of two sources: data on the activities of the courts and the daily regional press. Paris is the national capital region of France, but also, and by far, the largest metropolitan area in France and can only be compared with two or three other metropolitan regions in Europe. Around the highly urbanised core composed of Paris and its suburbs, a peri-urban belt has received many residential and industrial activities that produce more or less urbanised rural landscapes. Within this peri-urban belt, the scarcity of well-located vacant spaces (well-connected to transport facilities and services centres) and the diversity of actors and interests that share the same rural environment raises many tensions and conflicts over farmland uses. But the peri-urban belt is also an area where several local development and planning initiatives dedicated to farmland protection and farming enterprises survival are currently carried out. So the level of conflict is quite high, and the local inhabitants are somewhat dissatisfied with decisions of building infrastructures of different types (highways, railroads, airport, windmills, new towns…) near the suburbs.

**Figure 2:**
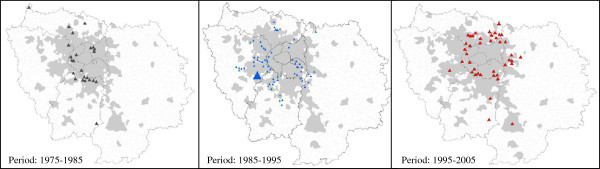
**Geography of infrastructure-related conflicts in the Greater Paris Region from 1975 to 2005 (litigation court sources).**

To start with, let us examine the geography of infrastructure-related conflicts^e^ in the Greater Paris Region, the size of the pyramids indicating the number of legal proceedings. These conflicts are a good indicator of the process of peri-urbanization the region has been undergoing, a process that is met with much opposition from populations that reside in the areas concerned. Figure 2 shows that the infrastructure related conflicts are not randomly distributed in the Paris Region. On the contrary, they are concentrated in the area bordering the urban centre of Paris: One can see that the highly urbanized part of the Paris metropolis (Paris and its three bordering departments, or the "*petite couronne*" – little crown) seems little affected. The conflicts are revealing of the spatial constraints the Paris agglomeration faces in its process of expansion and in the construction of the infrastructures that are necessary to the implementation of new urban developments.

The evolution of infrastructure-related conflicts over three successive periods - 1975–1985, 1985–1995 and 1995–2005 - corresponds to the expansion of the areas represented in grey on the map, areas which correspond to municipalities with a population of over 5000 inhabitants. Over the last thirty years, the grey area has not expanded much but that the conflicts have multiplied in different places, all located at the border of the “*petite couronne*”. They are peri-urban municipalities at the interface between the Paris agglomeration and the natural and agricultural areas which still represent over 50 percent of the total area of the Paris region. These municipalities have a high rate of urbanization (on average over 50 construction permits issued every year) and will no doubt become urban municipalities. The conflicts show that urban expansion does not always occur easily, because attempts to create infrastructures are confronted with organized opposition from residents who wish to preserve nature or their living environment. Conflicts here often take the form of opposition to decisions made by the public authorities, whether they concern the construction or the extension of infrastructure. They are a good expression of the process of legitimation of reflexivity that starts (Rosanvallon, 2008), in particular when it is based on logic of proximity with local populations exercising their democratic rights to intervene.

**Figure 3:**
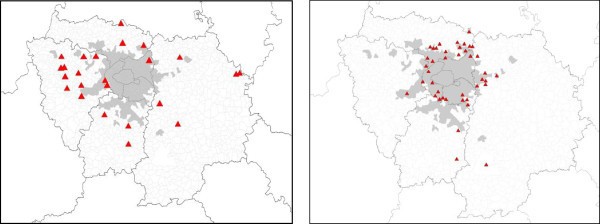
**Conflicts related to infrastructure of public utility reported by the Daily Regional Press (2005) and litigation rulings (1999–2005) in the Greater Paris Region.**

**Figure 4:**
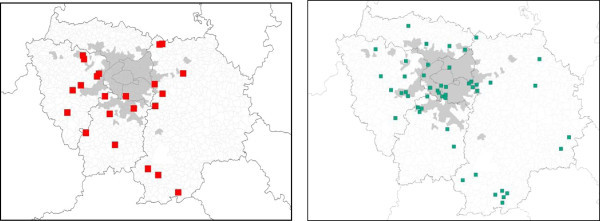
**Conflicts related to urban development and the development of open spaces reported by the Daily Regional Press (2005) and litigation rulings (1999–2005) in the Greater Paris Region.**

The *Conflicts* © database can also be used for examining the logics behind the location of conflicts such as they are revealed through the analysis of two different sources compared with the results of surveys conducted in this zone. For example, we observe heterogeneity between the DRP sources (with the daily newspaper "*Le Parisien*") and the litigation courts' data on conflicts related to infrastructures of public interest (Figure 3) or to urban development or the development of open spaces (Figure 4). The conflictual dynamics around the infrastructure identified by the local press clearly indicate that conflicts are far more intense in the western part of the Paris region where the value of land, the average income of the residents and associative activities are higher than in the rest of the region. Thus, mediatization, patent in the press articles, seems conditioned by a specific social context that gives conflicts a particular "territorial style". However, the similarities between the results obtained from both sources seem much stronger in the case of the conflicts related to urban development and the management of open spaces.

We can go further and undertake systematic comparisons between both sources, so as to compile complete and opposable profiles of conflictuality in the same zone. Figure 3, built from data covering the 2003–2005 period, provided by the DRP, highlights the different types of conflicts reported, as well as the number of press articles published about them. This gives us an idea of the intensity of those conflicts, as well as their respective repercussions. A distinction is made here between remedial conflicts (which start after the implementation of an infrastructure for example, or the occurrence of a contested action) and preventive conflicts (which are triggered when a project is publicized, for example, in the framework of a public enquiry pertaining to a building permit), a distinction that is possible thanks to the fact that the data have dates associated to them.

**Figure 5:**
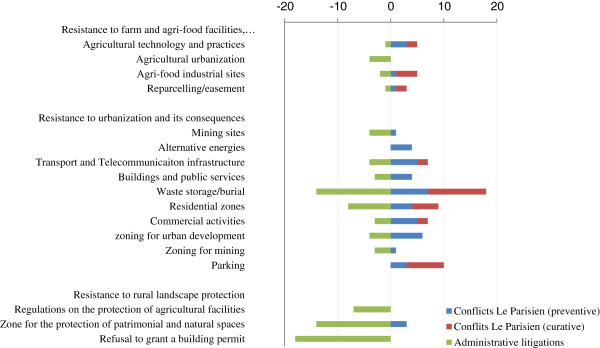
**The main sources of land use conflicts in the Greater Paris Region, according to the administrative courts' rulings (Versailles, Cergy, Melun) (Source:** ***Le Parisien*** **, 2003–2005; Archive collections of Administrative tribunals, 2005–2006); Darly** 2009 **.**

A process similar to that conducted with the DRP can be undertaken using the decisions made by tribunals, and more particularly by administrative tribunals. This leads to noticeably different results (Figure 5), which reveal the importance of using different sources of data for analyzing conflicts.

**Figure 6:**
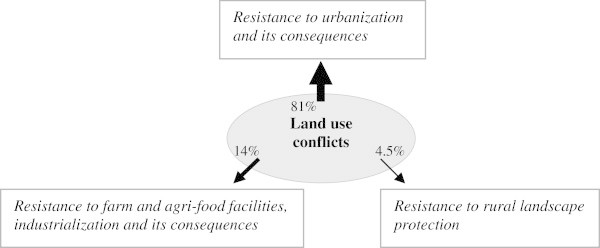
**Conflict in the Greater Paris Region as observed by the DRP (Source:** ***Le Parisien*** **, 2003–205); Darly** 2009 **.**

The database can also be used for analyzing more precisely certain categories of conflicts - in this case the conflicts related (directly or indirectly) to the uses of agricultural land (Darly 2009; Darly and Torre 2013a, b). We make a distinction between three main groups of conflicts according to the characteristics of the contested facilities: the facilities dedicated to the functioning of the city – construction of transport or energy infrastructures, productive or residential activities, burial or spreading of waste, building and housing, commercial and industrial zones – the mechanisms related to agricultural economics in these territories - easements, re-parceling, and those related to landscaping projects – zones for the protection of land and natural resources, Regional Natural Parks – (Figure 6). Here again, we can see the importance of the conflicts related to the presence of the capital city (Paris) and to its expansion into peri-urban areas which regularly encroaches on land areas reserved for agricultural purposes or nature conservation and provokes the opposition of some of the people already residing in these areas.

**Figure 7:**
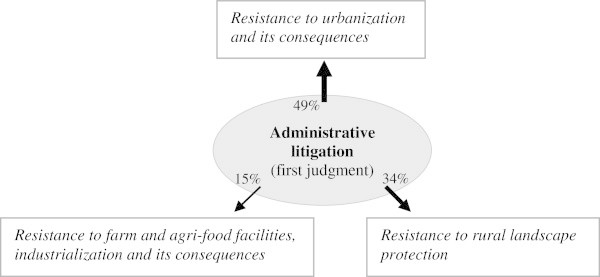
**Conflict in the Greater Paris Region as described in legal cases dealt with by the Administrative Appeal Courts (Versailles and Paris) and the Council of State (1981–2005); Darly** 2009 **.**

Comparing the results of the analysis of the DRP and of litigation ruling (Figure 7) shows noticeable differences in the case of conflicts related to the use of agricultural land: while the oppositions against the regulatory mechanisms for the protection of nature and open spaces represent a large number of litigation cases. The press reveals, more specifically, the collective and publicized dimension of the actions undertaken against so called urban sprawl interest projects (against infrastructures for example). This finding indicates that certain conflictual situations seem to lack the public dimension necessary for their publicization and therefore hardly feature in the press, but take the form of individual litigation cases. These cases mostly involve oppositions against administrative decisions such as land occupancy authorizations in urban law (building permits and planning certificates for example). Finally, a number of conflicts that have been the object of litigation concern similar categories of actors: It is the case of oppositions against some land restructuring projects, internal to the farming world. Inversely, it is mostly the confrontation between local residents and nonresident users that appears to be the main source of the conflicts that feature in the local press, conflicts in which the collective action dimension prevails.

### Conflicts in developing countries. An example in South Pakistan

**Figure 8:**
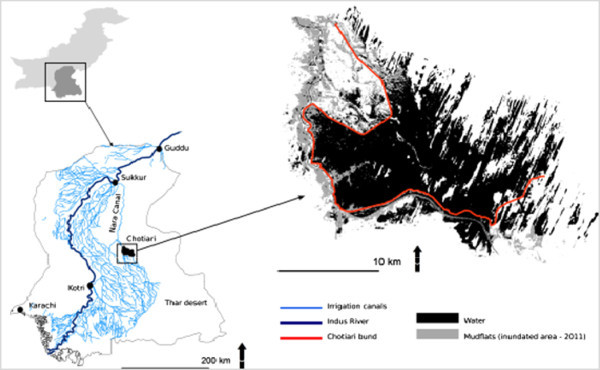
**Location of the Chotiari water reservoir.**

For a particular case study of land use conflict in developing countries, we have selected the case of Chotiari water reservoir from Pakistan; in order to put light on the land use conflicts caused by an infrastructural project setting with follow-up governance structure. This is one of Pakistan’s largest infrastructure projects, which is facing opposition in the country and is held up as an example of weak governance in the planning of new infrastructures in developing countries. (Magsi and Torre, 2014) (see Figure 8).

The Chotiari reservoir project was designed and implemented in order to increase the storage capacity of the existing lakes in the Chotiari wetlands and enable the irrigation of more arable land in Pakistan. The project was initiated in 1992 by the Water and Power Development Authority (WAPDA) and was funded by donor agencies via the World Bank. The project area extends over 18,000 hectares of entitled and unentitled land. The Chotiari reservoir area was characterized as wetlands and included lakes, forest, swamps, irrigation channels, agricultural land, barren land and a rich ecosystem, which supported the livelihoods of the local population through fishing, agriculture, grazing and a range of other economic activities. The reservoir project has created opposition between the principal actors (fishermen, farmers, livestock herders and others) on the one hand, and stakeholders from the public administration (national and provincial ministries), local politicians and landlords on the other. More specifically, a number of factors have made the task of implementing this project more complicated and controversial: the public administration’s highly bureaucratic approaches and mismanagement of construction and compensation funds; local politicians’ misuse of position and power with regard to forced displacements; and local landlords’ exercise of power over the local population. Furthermore, opposition grew when local populations were dispossessed of their livelihoods and ancestral properties without proper compensation. In spite of all these issues, the public authorities completed and inaugurated the reservoir in February 2003, five years later than anticipated.

**Figure 9:**
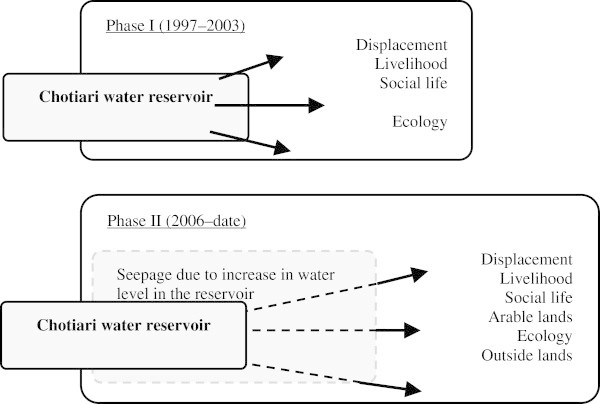
**Conflict dynamics of Chotiari reservoir.**

For this case study we have collected data through various sources, i.e. DRP, experts' opinion interviews by an open questionnaire and other sources (available literature, GIS and internet). The Chotiari project results reveal that local actors and outside stakeholders are under opposite aims and objectives of land use, and that the drivers of this situation (behaviors and interpersonal relations and actions) lead the project under situations of superposition of uses. This restlessness among stakeholders encouraged local journalists to demonstrate their issues. In fact, more than eighty percent of the articles (of total published news/articles in DRP since 1997–2011) reflected that there has been significant wrong doing associated with the land acquisitions, compensation and resettlement plans. Therefore, the project has brought on the dynamics of conflicts of land use, which has not only contributed in the changing of territoriality of the actors, but also came forth with disruption of socio-spatial practices. This interference of socio-spatial practices involved a reaction of discontent, which has sometimes expressed aggressively (Iqbal 2004). Moreover, public authorities have induced social and environmental nuisances by affecting arable lands, pasture, forest, as well as cruel displacement of local population. Besides all, the increasing water level in the reservoir is creating seepage^f^, which is destroying adjacent agricultural lands (see Figure 9).

The multi-dimensional catastrophe of the Chotiari reservoir cannot be understood with a single factor. Therefore, it is important to visualize and quantify the structural and proximate factor dynamics with their anticipation, which have not only escalated conflicts of land use but also unrest among local population. Therefore, on the basis of articles published in daily press and opinions interviewed from experts, we came into effect to disclose the responsible factors to the conflicts of Chotiari reservoir (see Table 1). In this regard we have quantified the factors which appeared in DRP as well as in the expert opinion interviews. These factors seemed responsible for either giving favorable path to pre-conditions or conducive climate to the conflicts.

**Table 1:** **Conflict factors of Chotiari reservoir**

Factor types	Cause 0073	Percentage	
		DRP	Experts opinion
Structural factors	Corruption/misuse of funds	23.94	34.38
	Unilateral decision	21.81	21.88
	Lack of technical and scientific research	19.68	9.38
	International interest	7.98	12.50
	Non-existence of national resettlement policy	9.04	9.38
Proximate factors	Ethnic diversity and disarray (unrest among communities)	13.83	12.50
	Others (Nepotism, Illiteracy etc.)	3.72	0

**Figure 10:**
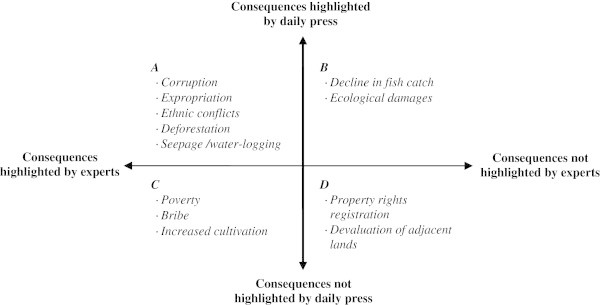
**Consequences of Chotiari conflict: by daily press, experts and personal observations.**

In the above table we see the differences in the factors of conflicts highlighted by either source, which may be due to the technical approaches and scientific analysis of both sources as well as the public character of DRP data compared with the privacy of face to face interviews. Therefore, we disclose the consequences (either positive or negative) of the project after deep analyzing of DRP as well as experts' opinion in the study area (see Figure 10). After comparative analysis we found both similarities and dissimilarities in the data sources, due to privacy or the public character of their approaches.

**Figure 11:**
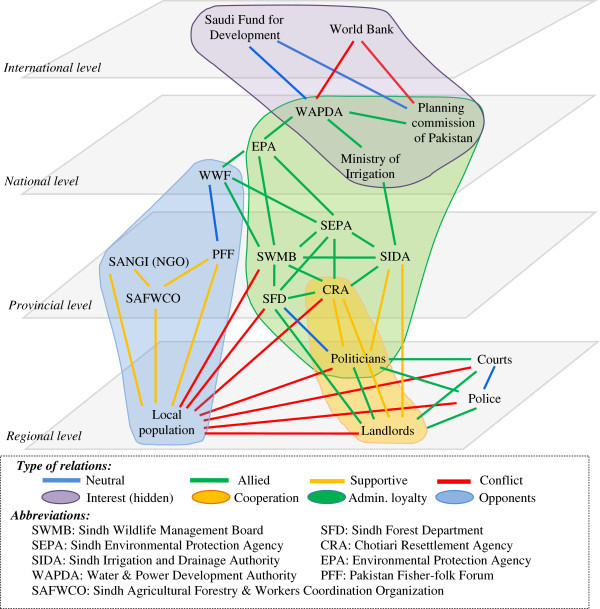
**Networks of local actors in the Chotiari conflict.**

Results of this case study present the pattern of thought based on the expert opinions, daily press and interviews of affected households in the study area which insights a simple approach towards social representations in the categories of the actors. Thus it is difficult to categorize from planning to construction stages of the project. For example, during policy making process the actors were involved either from regional to national level, while some actors involved temporarily and did not play an active role in the administration. In order to understand the dynamic process of the project, we have tried to analyze a relational approach to provide some answers regarding social representations corresponding to a universe of interrelated elements. Though, after deep analyses we have summarized the relations, links and locations of the actors or stakeholders at different geographical scales (see Figure 11).

Above figure exposes the network of the actors involved in Chotiari reservoir, with different relations and behaviors. The results show that those stakeholders have categorized themselves in defined strategies of construction and opposition of the reservoir (networks of pros and cons). Dramatically, the managing and administering actors (international to national scale) have a different representation on the reservoir area; they were found in alliance with the local politicians and landlords (regional scale), in order to construct the reservoir. According to the experts the cooperation of local stakeholders with administrative actors from international to national scales was based on some hidden interests of corruption and favoritism, while landlords and politicians have supported for construction reservoir. In this figure it is also disclosed that between two pro-construction networks (initiators and supporters), an administrative loyal network have played a key role to construct the reservoir. On the other hand, local actors were found in protestation against reservoir construction, relocation and compensation issues (Magsi and Torre, 2012). While, local population with the support of different NGOs have opposed decision of reservoir and manifested to protect natural resources of precious wetlands of Chotiari. Their limited scale of support led no valuable results of their oppositions, protests and agitations, because despite of this the reservoir has been constructed and inaugurated on February 2003. Results related to multilevel governance, we may say that this drama may be played to divert people’s attention that conflicts are at regional scale only. Moreover, through above figure we have explored that law enforcement institutions (courts and police) are peripheral and seem more suppressive rather to have influence on the administration, in this situation the local population will surely have no hope of their violated rights. Through this case study we acquire the information on infrastructural development settings without agreement of local stakeholders. In fact, the only way to examine the institutional inconsistencies and distribution of dissimilar power, leading to land use conflicts and loss of local population’s resources, is to analyze the dynamics of actors/stakeholders network in the study area, such as the reaction of all stakeholders during and after the project construction.

## Conclusions

This article has aimed to present the work carried out in the last few years by a multidisciplinary team on the question of land use conflicts and to reveal the methodology of survey and data collection, as well as the structure of the resulting database. We have first presented the scope of our investigations by defining the conflicts in question, their characteristics, motives, manifestations and the actors involved. We have then presented our method of identification of conflicts, based on a diagnosis of the conflicts that occur in selected areas and on the combination of various methods of data collection, including interviews with experts, analyses of the DRP and of litigation rulings. Lastly, we have presented the *Conflicts* © database, with its tables and nomenclatures, which reconcile the data collected from different sources, before providing a few examples of how we have used implemented our method with cases of conflicts occurring in the Greater Paris Region.

Our studies, performed on the basis of the previous method, reveal that land use conflicts are characterized by a high diversity of expression depending on the activities, on the uses around which they emerge, on the territories in which they occur, as well as on the characteristics of the actors involved in the conflicts in question. Furthermore, some conflicts, which are closely related to certain specialized activities, are kept relatively private and may even be limited to face to face interactions between two private parties, whereas others, related to decisions concerning a large number of individuals and involving questions of land use regulations, are liable to involve the participation of the public authorities and associations representing part of the population (for example conflicts related to the definition of local urban development plans or of protected zones). The quantitative survey, crossing various sources of information, and the qualitative surveys show that land use conflicts can take extremely diverse forms of expression and manifestations, but are however centered on large categories of conflicts, territories or modes of resistance to undesirable projects. Thus, observing conflictuality has nothing to do with the mere collection of raw information expressing a reality that is easy to decrypt. The goal of the crossed analysis of various information materials is precisely to explain the fact that the modes of expression of conflicts do not just constitute "a source of information" but a framework of observation that determine the types of phenomena observed.

Our research into conflicts in rural and periurban areas shows that this dimension is key in processes of territorial management, regional development or the governance of various local activities. Sometimes, conflicts are blind oppositions or are the product of egoistical Nimby behaviours. But in many cases they constitute a way of initiating discussions on the issues and paths of territorial development and of influencing decisions by participating in processes underway from which one had been excluded. That is why they have a bearing, either on the decisions on land use and management (arbitrated negotiation) or on the composition and representativeness of the bodies responsible for taking decisions (arbitral negotiation). The conflict thus becomes an integral part of the deliberative process at the local level by allowing an expression of local democracy and the re-inclusion of participants who were forgotten or deliberately excluded during earlier project development stages.

Land-use conflicts thus constitute one form of resistance and expression of opposition to decisions that leave part of the local population unsatisfied. Some local innovations, whether technical or organizational in nature, give rise to resistance which can turn into conflict. Major changes requiring reconfiguration of the use of space (creation of transport, energy or waste-processing infrastructure, new urban master plans, territorial or environmental zoning, etc.) generate conflicts whose spatial and social extent can quickly grow. Conflicts are signals of social, technological and economic changes, indicators of novelty and innovations. They demonstrate the opposition aroused by the latter, lead to discussions on their implementations and their possible (non-) acceptability as well as on the adoption of governance procedures and their transformation under the influence of the dynamics of change. All changes encounter opposition or resistance of varying relevance and justification. But it would however be simplistic to see this resistance as a systemic sign of reactionary opposition to change because, in a number of cases, they are more a reflection of differences over the direction taken by the new initiatives that are being imposed on the public than of a stubborn desire to maintain the status quo. During these phases of conflict, social and interest groups tend to reconstitute themselves and may even undergo technical or legal changes. Once a conflict ends, it leaves behind new local agreements, new modes of governance, new configurations of discussion forums as well as new technical procedures (changes in direction, various adjustments, changes in urban planning documents, etc.), all arrived at during the negotiations.

Territorial governance processes are today undergoing intense upheavals and are subject to intense periods of discussions and conflict oppositions. These latter shape the phases of territorial innovation and thus change the directions of development and growth in rural or urban territories. Such governance mechanisms and their conflict sides can be viewed as laboratories of change because they accompany and sometimes anticipate the changes underway in the territories by giving them shape, by helping maintain a dialogue and expressions of opposition and by preventing violent confrontations or failures of development due to sluggishness or expatriation. Therefore, these changes in land use occupations and the subsequent oppositions they gave birth to are embodied in the opposing and twin forms of conflict and consultation which constitute the modes of expression and the vehicles of transmission of on-going innovations at the territorial level.

### Endnotes

^a^For example, in Pakistan tribunals refused to provide data on land use conflict related cases because of involvement of bureaucrats, feudal and politicians.

^b^One can associate to each conflict the list of municipalities in which are located the sites that are at the center of the conflict.

^c^The data contained in the data base are accessible to the interested readers if they want to contact the authors. The detailed structure of the data base is also available (Galman and the participants of Conflict Program 2007)

^d^The Seine Estuary, the Loire Estuary, the Regional Natural Park of Monts d'Ardèche, the Pays Voironnais in Isère, the Community of municipalities of Montrevel en Ain, the Cortenais, and Balagne areas in Corsica, the Puys mountains in Auvergne, the Réunion island area, the Greater Paris Region, the Charente catchment area, Montpellier's coastline, and Arcachon Bay.

^e^They concern, for the most part, the projects of construction of road, highway, railway, river or airport infrastructures, as well as public facilities such as wastewater treatment plants, town halls, barracks, prisons, multipurpose halls…

^f^The organizational management structure has allowed increasing water level in the reservoir for which purpose it has been constructed, but due to low standard earth work there is water seepage from embankments which is damaging agricultural lands outside the reservoir.
